# Supplementary figures and images for: Exendin-4 enhances GLP-1 signaling and reduces anxiety-like behaviors in male heroin withdrawal mice
Source: PLoS One. 2026 Mar 12;21(3):e0343995. doi: 10.1371/journal.pone.0343995 (PMC12981496; doi:10.1371/journal.pone.0343995)

**Fig 4.** .....

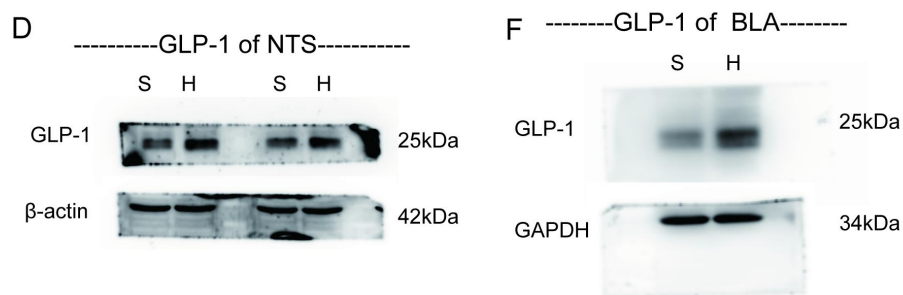

**Fig 6.** .....

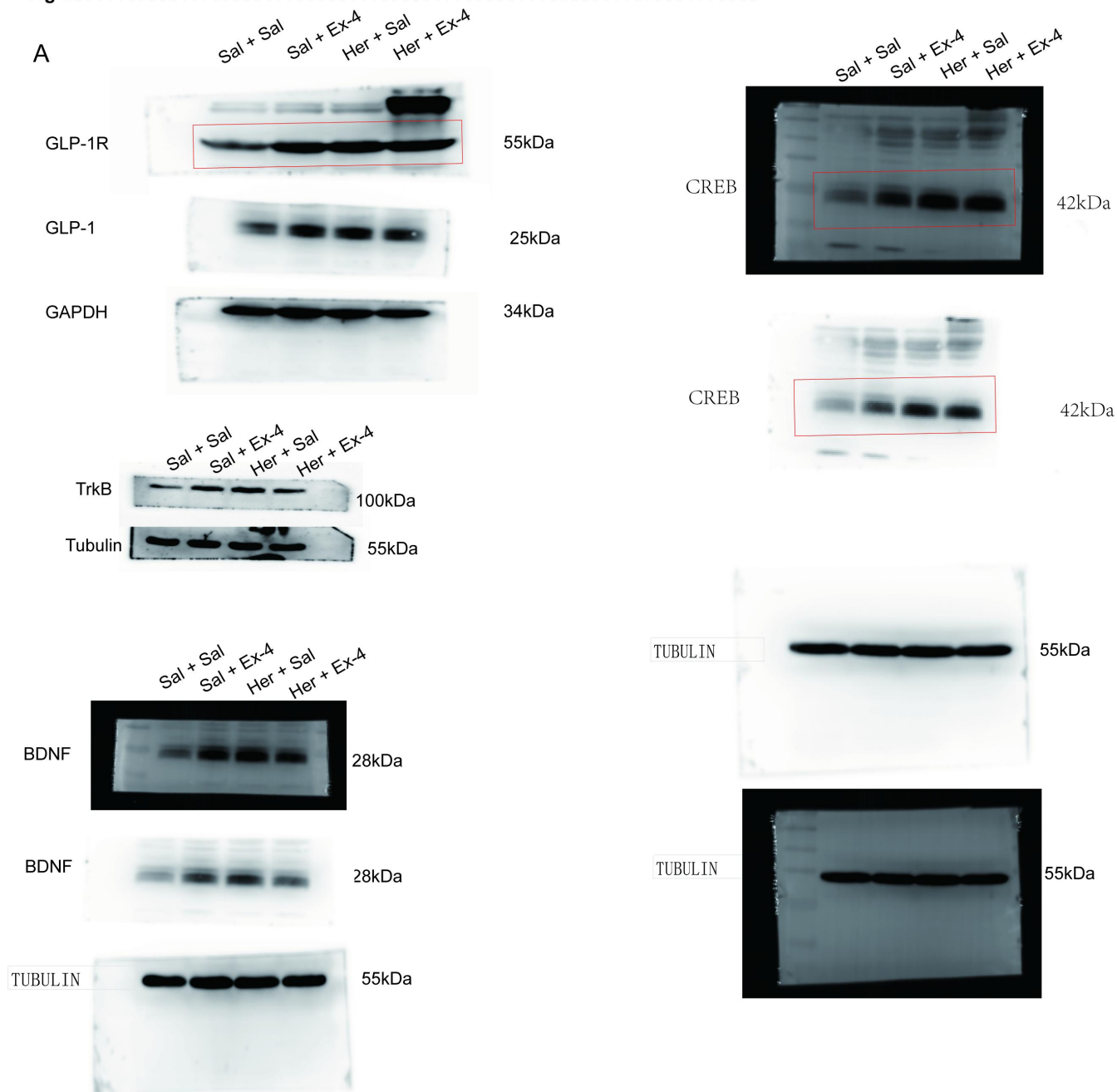

Supplement: S1 Raw Image — (PDF) [file pone.0343995.s001.pdf]
