# Supplementary material for: Exendin-4 enhances GLP-1 signaling and reduces anxiety-like behaviors in male heroin withdrawal mice
Source: PLoS One. 2026 Mar 12;21(3):e0343995. doi: 10.1371/journal.pone.0343995 (PMC12981496; doi:10.1371/journal.pone.0343995)
Supplement: S1 File — (DOCX) [file pone.0343995.s008.docx]

1、Blood glucose test

Utilize the following procedure to measure the blood glucose level in a mouse: Secure the mouse in an appropriate fixture and expose its tail. Clean the tail using an alcohol swab. Employ sterilized ophthalmic scissors to make an incision on the tail for blood collection, ensuring the initial drop of blood is wiped away. Insert a blood glucose test strip into the blood glucose meter, and apply a sample of blood from the mouse's tail to the test strip. Record the blood glucose reading. Perform this measurement twice more, and calculate the average of the three readings to determine the mouse's blood glucose level.

2、Retrograde Tracing Technique

(1)Stereotactic Positioning: Following the administration of an intraperitoneal injection of avidin solution and the induction of anesthesia in mice, a suitable depilatory cream should be applied to the scalp using a cotton swab. After allowing the cream to sit for a few minutes, it should be thoroughly wiped off with filter paper. The area is then disinfected with iodine solution, followed by cleansing with a cotton swab soaked in disinfectant alcohol to remove any residual iodine. The mouse's head is secured in a stereotaxic apparatus, and erythromycin eye ointment is applied to protect the eyes. An incision is made along the midline of the scalp using a surgical scalpel, and ophthalmic forceps are employed to separate the skin along this line. The connective tissue on the skull is cleaned with a cotton swab, and brain localization is performed. The coordinates for basolateral amygdala (BLA) injection are as follows: anterior-posterior (AP): -1.46 mm from Bregma; mediolateral (ML): 2.7 mm; dorsoventral (DV): 4.5 mm. A skull drill is used to access the specified location.

(2) Stereoscopic injection: Inject 0.6μL of pAAV-hSyn-EGFP-2A-Cre-WPRE virus into the BLA brain area using a glass electrode needle at a rate of 0.1μL/5 minutes.

(3) After injection, slowly withdraw the needle, suture the scalp, and place the mouse on a heating pad to rest.

(4) For the first three days, penicillin was applied to the wound every day to prevent the mice from getting sick. The mice were continued to be housed for 18 days, waiting for the virus to be traced and expressed in reverse. Then, the samples were fixed and frozen sections were made. After staining the DAPI cell nucleus, images were collected using a confocal laser microscope.

3、Western blotting

Mice were anesthetized using 1.25% avidin, followed by euthanasia via cervical dislocation. Subsequently, the entire brain was excised and immediately frozen on ice. For the Western Blot (WB) experiment, the basolateral amygdala (BLA) and nucleus tractus solitarius (NTS) brain regions were isolated, weighed, and subjected to protein lysis buffer preparation. Proteins were extracted from the lysates, quantified using the bicinchoninic acid (BCA) assay, and standardized across samples. This procedure was repeated four times to ensure the consistency of internal reference proteins, such as GAPDH, prior to the detection of the target protein. Proteins were then separated via SDS-PAGE and transferred onto a polyvinylidene difluoride (PVDF) membrane. The membrane was blocked with 5% skim milk for two hours, followed by incubation with primary antibodies (GLP-1R, GLP-1, BDNF, TrkB, CREB) at a dilution of 1:1000, and GAPDH at a dilution of 1:2500 overnight. Subsequently, the membrane was incubated with a horseradish peroxidase (HRP) conjugated secondary antibody (diluted 1:2500 in TBST) at room temperature for 1.5 hours. Immunoblotting was visualized using enhanced chemiluminescence, with image acquisition conducted via an exposure machine, and grayscale values were analyzed using ImageJ software.

4、Immunofluorescence staining

Anesthetize the mice using 1.25% Avedin at a dosage of 0.2 ml per 10 g of body weight. Proceed to dissect and expose the heart, followed by perfusion with physiological saline and 4% paraformaldehyde via a syringe. Once the lung and liver tissues have been adequately perfused, proceed to collect the brain tissue. Fix the brain tissue in 4% paraformaldehyde for a minimum of 24 hours. Subsequently, dehydrate the tissue in a graded ethanol series, render it transparent using xylene, and immerse it in a semi-benzene semi-wax or wax solution prior to embedding in paraffin. Trim the paraffin blocks and section them into slices with a thickness of 6 μm, specifically collecting sections that contain the basolateral amygdala (BLA) brain regions. Affix the slices onto glass slides and bake at 38°C for 48 hours. Immerse the slices in xylene and anhydrous ethanol twice for 10 minutes each, then immerse them sequentially in 95%, 85%, and 75% ethanol for 5 minutes each. Rinse the sections three times with phosphate-buffered saline (PBS) for 15 minutes each. Incubate the sections with a 5% goat serum solution at room temperature for 30 minutes. After removing the goat serum solution, apply the primary antibodies at specified dilutions: GLP-1R and c-Fos at a ratio of 1:100, and VGLUT1 and GAD1 at a ratio of 1:200, and incubate overnight at 4°C. On the second day, the tissue slices underwent three washes with PBS, each lasting 15 minutes. Subsequently, they were incubated with a fluorescent secondary antibody (488/CY3, 1:500 dilution) at room temperature for 120 minutes in the dark. Following incubation, the slices were again washed three times with PBS for 15 minutes per wash. They were then fixed using an anti-fade mounting medium containing DAPI and sealed with nail polish. High-resolution spinning disk confocal microscopy was employed to capture images of the samples. Fluorescence intensity and cell counting were analyzed using ImageJ software.

5、Open field test (conducted between 9am and 6pm, with lighting level maintained at 100 lux)

(1)Transfer the mice from the animal holding room to the testing environment, ensuring that the ambient temperature is consistently maintained at 25°C. Activate the white noise generator to mask external sounds, taking care to avoid excessive volume. Allow the mice to acclimate to the testing room for a minimum duration of 30 minutes prior to commencing the experiment.

(2)Carefully grasp the tail of a mouse and remove it from its cage. Position the mouse in the central area of the open field apparatus, which consists of a four-unit maze with each unit measuring 50 cm in length, 50 cm in width, and 45 cm in height. A 25 cm x 25 cm square is delineated in the center of the open field.

(3) Allow mice to move freely in each quadrant of the open field for 5 minutes, while using the Tracking Master V5.3.7 system to collect mouse movements. Record the number, time, and distance of mice entering the central area of the open field as indicators of anxiety like behavior. Each mouse undergoes one experiment in our test. If there are significant individual differences in the test data, a second test may be considered, but the interval should be at least 30 minutes.

(4) After the testing phase is over, gently pick up the mouse, remove it from the maze, and then put it back in the cage.

(5) Remove all feces from the open area, wipe all urine, spray 50% ethanol on the floor and walls of the maze, and wipe clean with a clean tissue.

(6) Then continue with the next mouse, repeat steps 2-5 above, use the Tracking Master V5.3.7 system to calculate the time, distance, and frequency of the mouse entering the central area of the open field, and finally perform statistical analysis.

6、Elevated Cross Maze Test (conducted between 9am and 6pm, with lighting level maintained at 100 lux)

(1) Transport the mice from the animal facility to the behavioral testing room, ensuring that the room temperature is maintained at approximately 25 ℃. Activate the white noise generator to mask external sounds, ensuring the volume is kept at a moderate level to prevent auditory stress. Prior to commencing the behavioral assessments, allow the mice to acclimate to the testing environment for a minimum duration of 30 minutes.

(2) A mouse was positioned in the central area of an elevated plus maze, oriented towards the closed arm. The apparatus used for this elevated maze assessment comprises two open arms, each measuring 25 cm in length, 5 cm in width, and 0.5 cm in height, arranged perpendicularly to two closed arms, each measuring 25 cm in length, 5 cm in width, and 16 cm in height. The central platform measures 5 cm by 5 cm. The open arms feature a minimal wall height of 0.5 cm to prevent the mouse from falling, whereas the closed arms are enclosed by walls measuring 16 cm in height. The entire apparatus is elevated 50 cm above the ground within an empty circular water tank, typically used for water maze testing. A blue cloth barrier encircles the water tank to deter escape attempts by the mice and to minimize the influence of environmental variations. The device is constructed from acrylic material, with a white platform and opaque walls.

(3) Mice were permitted unrestricted movement within each arm of the elevated cross maze for a duration of 5 to 10 minutes. During this period, the Tracking Master V5.3.7 system was employed to monitor and record the mice's movements, specifically noting the frequency, duration, and distance of entries into the open arms. These metrics served as indicators of anxiety-like behavior. Each mouse participated in a single experimental trial. In instances where substantial individual variability was observed in the test data, a subsequent trial could be administered, provided a minimum interval of 30 minutes was maintained between tests.

(4) After each test, clean and wipe all arms and central area with 50% alcohol to remove feces and urine left by the mice, and then proceed to the next round of testing, repeating steps 2-3 above.

(5) The distance, frequency, and time of all mice entering the open arm were calculated and analyzed by the Tracking Master V5.3.7 system, and then statistically analyzed.

7、Forced swimming test (conducted between 9am and 6pm, with lighting level maintained at 100 lux)

(1)Transfer the mice from the animal room to the testing room, where the temperature is maintained at 25°C, and allow them to acclimate for a minimum of 30 minutes prior to commencing the test.

(2) Fill a transparent acrylic cylinder, measuring 30 cm in height and 20 cm in diameter, with tap water. Adjust the water depth according to the size of the mouse to ensure that its hind legs cannot reach the bottom of the container. For consistency, a uniform water depth of 20 cm was selected, and the water level was marked on the cylinder to ensure uniformity in subsequent trials.

(3) Conduct the forced swimming test using tap water maintained at 25°C. The water temperature should be verified with a thermometer to ensure it remains within the range of 25 ± 0.5°C for testing purposes.

(4) Initiate the Tracking Master V5.3.7 recording and analysis software and position the camera optimally to achieve the highest possible resolution of the subject animal. Subsequently, place each mouse in a water-filled cylinder for a duration of 2 minutes, followed by a 4-minute recording of the mice's forced swimming behavior using the Tracking Master V5.3.7 software.

(5) Upon completion of the 4-minute recording, carefully remove the mice from the cylinder, gently dry them with a towel, and transfer them to a temporary enclosure lined with sawdust. Utilize an electric blower to facilitate the drying of the mice's fur. During their recovery in the cages, it is imperative to maintain close and continuous monitoring. Furthermore, it is advised that not all cages be positioned in proximity to indoor heaters; once the majority of the fur is dry, the mouse cages may be relocated to a more distant area.

(6) After each test, replace the water in the cylinder to prevent any potential effects on subsequent subjects, and then proceed with the testing of the next mouse, repeating steps 2-6 as necessary.

(7) Finally, use Tracking Master V5.3.7 software to analyze the 4-minute stationary time.

8、Tail suspension test (conducted between 9am and 6pm, withlighting level maintained at 100 lux)

(1)Transfer the mice from the animal housing facility to the testing room, ensuring that the ambient temperature is maintained at 25°C. Allow the mice to acclimate to the testing environment for a minimum duration of 30 minutes prior to commencing the experiment.

(2) Initiate the Tracking Master V5.3.7 recording analysis software and position the connected camera optimally to ensure maximum resolution. Prepare to document the tail suspension activity of the mice.

(3) Encase the base of each mouse's tail with a plastic hose slightly larger than the tail itself, serving as an anti-climbing device. The hose should measure between 2 cm and 3 cm in length; a shorter length may fail to prevent climbing, while a longer length may hinder subsequent tape application. It is advisable to utilize the middle section of a Babbitt straw to fabricate the anti-climbing device.

(4) The adhesive tape should be affixed at the distal end of the mouse's tail, allowing for a 2-3 mm extension of the tape beyond the tail's exterior. The application of the tape must accommodate sufficient space for suspending the mouse, and it is essential that the tape is positioned with a specific curvature along the tail to effectively prevent the mouse from detaching during the hanging process. It is important to note that the tape wrapping for each mouse should be conducted individually prior to testing; uniform wrapping across all subjects may increase the risk of detachment during the experimental procedure.

(5) Subsequently, perforate the adhesive tape affixed to the tail of the mouse using a needle suspended from the upper section of the tail rack. Suspend the mouse and initiate a timer for a duration of 2 minutes. Following this, employ the software to record the mouse's activity for a subsequent period of 4 minutes.

(6) Upon completion of the experiment, return the mice to their cages and carefully remove the tape from each tail. Avoid tearing the tape off the tail, as this may inflict pain on the mouse.

(7) Clean any feces and urine beneath the tailstock, sanitize the instrument with 50% ethanol, and then proceed to the next round of testing by repeating steps 2-7.(8) After completing the tail suspension test on all mice, use Tracking Master V5.3.7 to analyze the resting time recorded for 4 minutes.

9、Mouse heroin dependence model :

The groups were compared for body weight, fur condition, and walking status to confirm successful randomization. Heroin dependence was induced using Bailey et al.'s method, with heroin injections given subcutaneously in increasing doses over ten days. The dosage schedule was 2 × 1 mg/kg on day 1, 2 × 2 mg/kg on days 2-3, 2 × 4 mg/kg on days 4-5, and 2 × 8 mg/kg twice daily on days 6-10. The control group received saline injections.

10、Test of withdrawal symptoms in heroin withdrawal mice

Heroin dependence was induced within a 10-day period. Following a 24-hour cessation of heroin administration, the mice were individually placed in transparent plastic containers with dimensions of 20 centimeters in diameter and 30 centimeters in height. Their activities were recorded using a camera for a duration of 10 minutes. During this observation period, the frequency of withdrawal-related behaviors, including standing posture, hair licking, face washing, and defecation, was documented and subsequently analyzed. The standing posture was defined as the mouse having both front feet on the ground, standing again, and then having the front feet on the ground once more; if no similar behavior occurred within the subsequent two seconds, it was recorded as a valid standing. If similar behavior was observed, it was recorded every two seconds. Hair licking was defined as beginning and ending with the mouse's tongue licking a specific area of hair, which was considered one effective lick. If the mouse changed positions significantly, such as moving from licking the hair on the left waist to the right side, it was considered a second lick ; The washing behavior of mice with an interval of more than two seconds between adjacent washing behaviors is considered as two effective washing behaviors, otherwise it is considered as one washing behavior; Defecation is counted based on the amount of feces produced by mice during defecation.
